# The effect of educational intervention based on the behavioral reasoning theory on self-management behaviors in type 2 diabetes patients: a randomized controlled trial

**DOI:** 10.1186/s12889-024-19207-0

**Published:** 2024-07-02

**Authors:** Fatemeh Ranjbar, Masoud Karimi, Elahe Zare, Leila Ghahremani

**Affiliations:** 1 Department of Health Promotion, School of Health, Shiraz University of Medical Sciences, Shiraz, Iran; 2 Research Center for Health Sciences, Institute of Health, Department of Health Promotion, School of Health, Shiraz University of Medical Sciences, Shiraz, Iran

**Keywords:** Diabetes, Self-management, Education, Behavioral reasoning theory

## Abstract

**Background:**

Diabetes self-management education is necessary to improve patient outcomes and reduce diabetes-related complications. According to the theory of behavioral reasoning, the likelihood of performing a behavior is predicted by the link between beliefs, motivation, intention, and behavior. This study aimed to investigate the effect of an educational intervention based on the Behavioral Reasoning Theory (BRT) on self-management behaviors in patients with Type 2 Diabetes.

**Methods:**

A randomized controlled trial based on BRT was conducted on 113 patients with type 2 diabetes, with a control group and an intervention group followed for 3and 6 months. Data were collected using a researcher-made demographic questionnaire based on the constructs of BRT and behaviors related to self-management in patients with type 2 diabetes. In the intervention group were provided, 8 sessions of diabetes self-management education based on BRT. The control group only received the usual training of the center. Data was analyzed using SPSS26 software.

**Results:**

After the educational interventions in the intervention group, there were statistically significant changes observed in the mean scores of all constructs, fasting blood sugar, and glycosylated hemoglobin. On the other hand, no statistically significant change was observed in the mean grades of the control group. All the observed changes were significant at the 0.05 level.

**Conclusions:**

The results of this study were in favor of the effectiveness of an educational intervention that promotes diabetes self-management behaviors, using the principles of the behavioral reasoning theory. Which can be used in the design of health promotion programs for patients with diabetes.

**Trial registration:**

Iranian Registry of Clinical Trials (IRCT), IRCT20131014015015N21.

**Supplementary Information:**

The online version contains supplementary material available at 10.1186/s12889-024-19207-0.

## Background

Diabetes is one of the most important problems of the health system all over the world, approximately 90 to 95% of these patients suffer from type 2 diabetes [1] and its prevalence and incidence is increasing all over the world [2]. Every year, more than 7 million people around the world are diagnosed with diabetes [3] and globally, it is the fifth cause of death in most developed countries [4]. It is predicted that the number of people with diabetes in the Middle East region will more than double by 2045 [2]. In terms of the total population of adults with diabetes, Iran ranks third in the Middle East [5]. According to the estimates of the World Health Organization (WHO), if effective measures are not taken to control and prevent this disease number of people with type 2 diabetes in Iran will reach more than 6 million by 2030 [6] and based on the annual growth rate, diabetes in Iran will reach the second place in the Middle East region [7].

Diabetes is a complex disease that requires daily self-management decisions by a person with diabetes [8].

Self-management diabetes is an active and dynamic process that often includes changes in lifestyle, including glucose management, diet, physical activities, stress management, medication adherence, foot care, and blood sugar health monitoring [9].

Diabetes self-management education (DSME) is a vital element in the care of all people with diabetes and is considered necessary to improve patient outcomes [10], which is a comprehensive combination of clinical, educational, psychosocial and behavioral aspects of care. It addresses the needs for daily self-management and provides a foundation to help all people with diabetes perform daily self-care with confidence [8] and has positive effects on diabetes knowledge, blood glucose control, and behavioral outcomes. It can prevent long-term complications such as eye and kidney complications, nerve involvement, cardiovascular diseases, and premature death [11].

The purpose of teaching self-management behaviors is to provide knowledge, skills and self-confidence to accept the responsibility of self-management to people with diabetes [12].

Self-management in patients with diabetes in terms of diet and exercise, frequent use of medications and blood sugar monitoring plays an important role in reducing diabetes-related complications and premature deaths and improves favorable results [13]. Diet and physical activity play the most important role in controlling and preventing complications in people with diabetes [14].

Self-management by people with diabetes is far less than optimal, as 12% of adults with type 2 diabetes do not follow self-management behaviors such as blood glucose monitoring, diet modification, physical activity at all, 60% to one or two behaviors, and only 28% of them complete self-management behaviors [15].

Education plays a major role in controlling and preventing diabetes complications [16]. Studies have shown that behavioral change educational interventions based on theory are very effective [17–19] and self-management educational interventions on patients with type 2 diabetes can significantly improve the attitude, knowledge of diabetes and other psychological variables of patients [3, 20] and adherence to medication and improving quality of life, and 90% of studies showed overall improvement DSME based of theory [21].

Behavioral Reasoning Theory (BRT) is a new theory and it can be seen as an improvement in the Theory of Planned Behavioral Control (TPB). BRT is related to several other behavioral theories, but offers different advantages or merits compared to them [22]. Theories such as the theory of planned behavior and the theory of rational action, which are mainly focused on the factors related to the acceptance of behavior and have ignored the resistance of people in implementing or opposing the behavior, have been criticized [23]. This theory has four main constructs of behavioral intention, attitude, reasons (for and against) and values​​ [24].

**Fig. 1:**
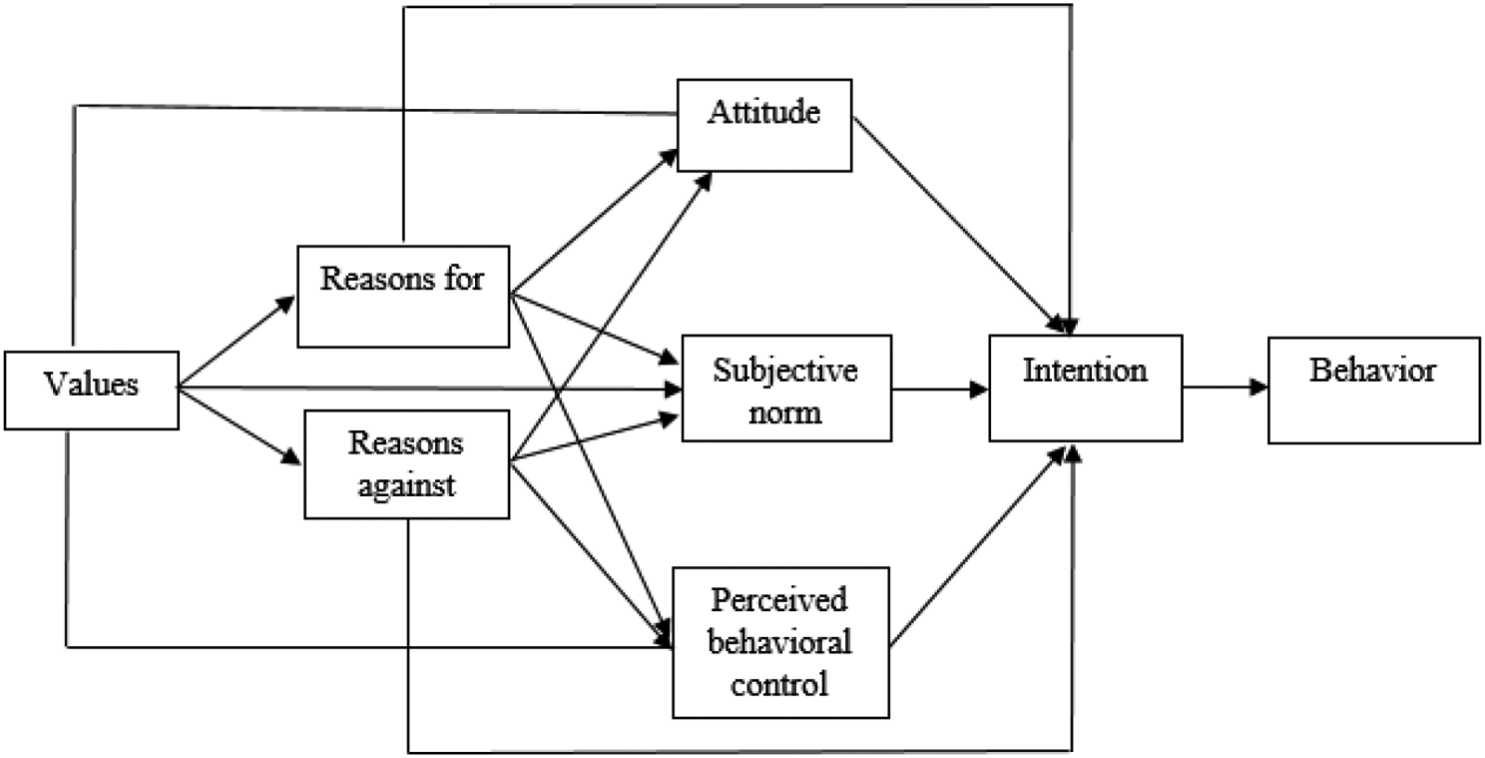
Behavioral reasoning theory

BRT proposes that reasons serve as important links between beliefs and motivation (e.g., attitudes, subjective norms, and perceived control), intention, and behavior. A basic theoretical assumption in this framework states that reasons influence motivation and intention [24] and provides important empirical links between values, beliefs and reasons (for and against), attitude, and behavioral intention [25]. Figure 1 [26].

During the planning and implementation of this study, the worldwide COVID-19 epidemic made it impossible to conduct face-to-face training. Therefore, a combination of virtual and face-to-face training was used [27]. Research has demonstrated that teaching self-management behaviors through virtual platforms can enhance healthy behaviors and help people control their blood sugar [28, 29].

Considering the high prevalence of diabetes in Iran, especially in Bushehr province, and the importance of self-management behaviors in the management of this disease, and in addition, to the prominent role of theories and models in correcting and improving health-related behaviors, this study aims to investigate the effect of an educational intervention based on the theory of behavioral reasoning on self-management behaviors in patients with type 2 diabetes was conducted for the first time in Iran.

## Methods

### Study design and participants

The study was intervention research conducted among 113 patients with type 2 diabetes who were receiving regular care and treatment at the comprehensive health centers of Bushehr in Iran in 2022. The research used a randomized controlled trial with two parallel arms and an equal allocation ratio to evaluate the effectiveness of the intervention.

The sample size is based on the study of Hailu et al. (2019) [30] and using the PASS NCSS software - version 15 with a confidence factor of 90% and a test power of 90% (Power = 0.9) and including 10% attrition. 60 people were calculated in each group.

To select the samples, we first chose four comprehensive health centers out of the ten available in Bushehr using a simple random method. The random allocation was done at the health center level, with two selected centers assigned to the intervention group (Quds Health Center and Meraj Health Center) and two centers to the control group (Kyber Health Center and Haft Tir Health Center). It’s important to note that the random allocation of comprehensive urban health centers was performed using the Consort checklist before individual recruitment. Next, 30 patients with diabetes who met the study entry criteria and came to receive healthcare were selected by a simple random method from each center. We used sequentially numbered containers to implement the random allocation sequence. Each container was labeled with a unique identification number, and participants were assigned to their respective groups by drawing a container from the set. We took steps to conceal the sequence until interventions were assigned: The person responsible for generating the random allocation sequence and preparing the sequentially numbered containers had no involvement in participant enrollment or assignment. They kept the list of allocations confidential until interventions were assigned. The research team members who designed and conducted the study generated the random allocation sequence. Enrollment of participants was done by healthcare providers at comprehensive health centers based on eligibility criteria determined by researchers. Participants were then assigned to interventions by drawing containers from sequentially numbered sets. Due to practical limitations, blinding of participants and healthcare providers was not feasible in this study. However, we took steps during data collection and analysis to minimize potential bias and the analyst was blinded.

In total, there were 60 people in the control group and 60 people in the intervention group.

To be eligible for participation in the study, individuals had to meet. specific criteria: a definite diagnosis of type 2 diabetes by a doctor, at least one year since the diagnosis, the ability to read, write, and speak Farsi, possession of a smartphone and proficiency in using WhatsApp, age between 30 and 60 years, and no severe complications caused by diabetes, such as eye disease, kidney disease, and leg/skin ulcers. Exclusion criteria included severe complications of diabetes during the study period, withdrawal from further participation in the study, death, and migration.

**Fig. 2:**
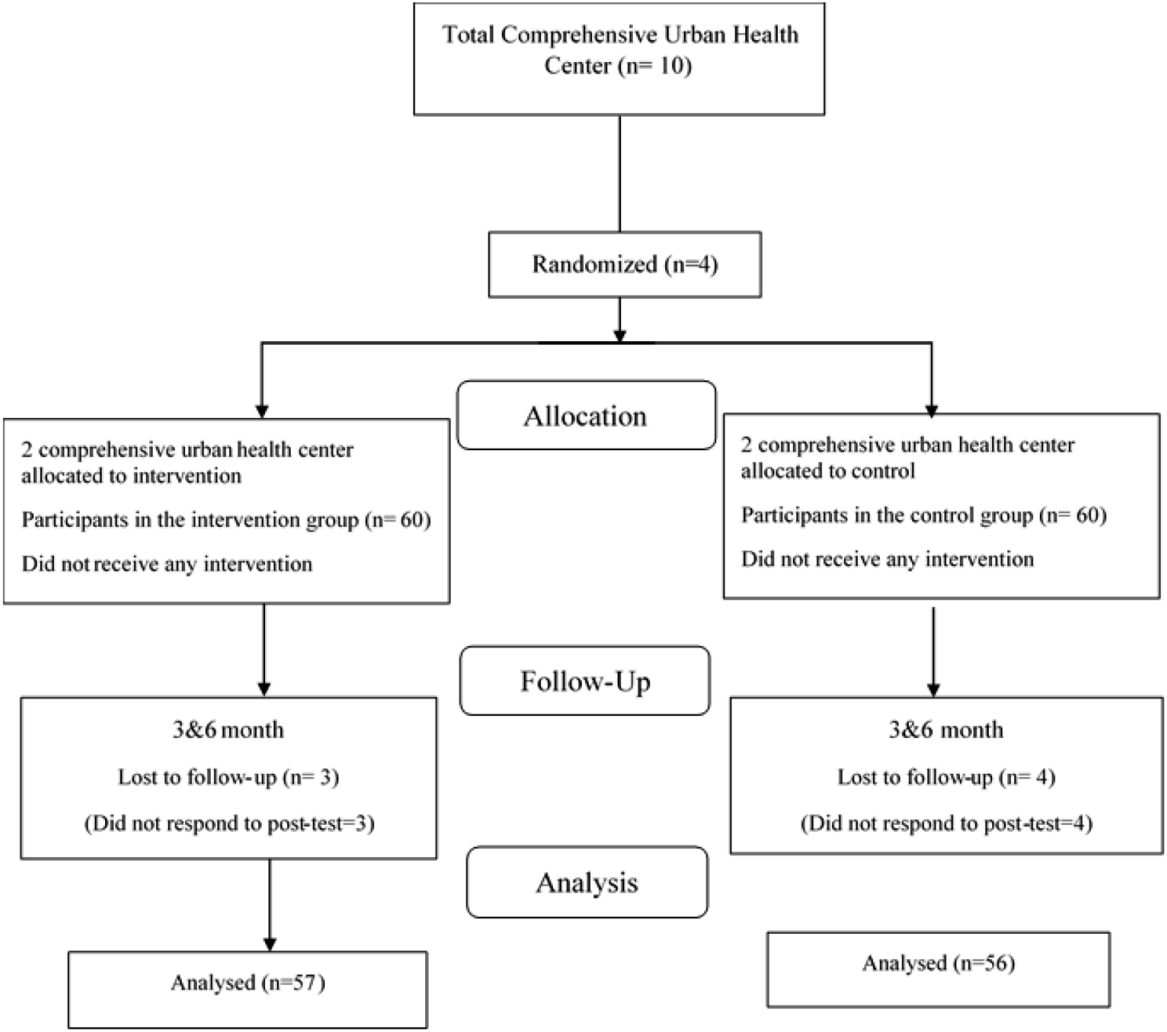
Consort flow diagram of participants through the study

After obtaining informed consent from participants and explaining the research objectives, both groups completed a pre-test questionnaire. Figure 2 indicates the flow chart of the present research.

### Measures

For this research, two questionnaires were utilized. The first questionnaire comprised of demographic information such as age, gender, education, marital status, occupation, and family history. On the other hand, the second questionnaire was a researcher-made one based on BRT constructs (Knowledge, attitude, intention, perceived behavioral control, subjective norms, reasons for, reasons against, and behavior) in the field of behavior change and implementing self-management behaviors (monitoring blood sugar, physical activities, adherence to medication, periodic examinations) in order to control and prevent short-term complications. The questionnaire used in this study was developed specifically for this research. An English-language version of the questionnaire is included as a supplementary file in the main manuscript. In addition to the behavior questionnaire, fasting blood sugar (FBS) and HbA1C were also measured as they can indicate behavior.

It is important to highlight that the primary outcome of this study was to assess the effects of various model constructs, including knowledge, attitude, intention, perceived behavioral control, subjective norms, and reasons for and against behavior change. The secondary outcomes measured were fasting blood sugar (FBS) and HbA1C levels. These measurements were taken at three months and six months following the intervention. HbA1C levels were determined using a biosystem kit and chromatography methods in a laboratory setting. It is worth noting that these biosystem kits are standardized and approved by the Ministry of Health, Treatment, and Medical Education in our country.

The questionnaire’s face and content validity were evaluated by a panel of experts and respected professors of health education. The CVI and CVR indices were calculated to ensure their validity. Additionally, the questionnaire’s internal reliability was measured using Cronbach’s alpha, and external reliability was evaluated through a retest method on a pilot sample of at least 30 people. Based on these evaluations, the questionnaire was deemed valid and reliable. The CVR for all constructs was greater than 0.78, indicating that it meets the acceptable criteria based on Lawshe’s standards [26]. Additionally, the CVI for all constructs was greater than 0.86, indicating that it meets the acceptable criteria based on Waltz and Bussel’s standards [31].

In the internal reliability test, Cronbach’s alpha coefficient was 0.7 for attitude, intention, and perceived behavioral control; 0.84 for behavior; 0.91 for reasons for and against, and 0.92 for subjective norms.

To measure the external reliability of a study, researchers retested 30 participants after two weeks. The researchers calculated the ICC intraclass correlation index for various constructs, including knowledge (0.94), attitude (0.84), intention (0.87), behavioral control (0.95), subjective norms (0.97), reasons for (0.97), reasons against (0.9), and behavior (0.92).

The participants in the study were subjected to three stages of pre-test measurement (when entering the study), three months and six months after the intervention.

### Procedure

People were invited to participate in the study after dividing the samples into control and intervention groups. Then, the study’s objectives were explained to the target group, and after assuring them of the confidentiality of the individuals’ information, informed consent was obtained from them. Next, fasting blood sugar and HbA1c of the patients were measured and in a group meeting, a demographic questionnaire and a researcher-made questionnaire based on the behavioral reasoning theory were completed by both groups.

The educational content was adapted according to national standards of diabetes self-care education (DSME) [13], including changes in lifestyle, glucose management, diet, physical activities, medication adherence, foot care, and blood sugar monitoring.

The intervention was developed by a medical doctor who had a PhD degree in Health Education and Health Promotion together with the research team consisting of an MSc student in Health Education and Promotion (first author). An MSc student in Health Education and Promotion (first author) conducted All educational sessions at comprehensive health centers.

The two-month training program consisted of eight face-to-face training sessions, held once a week. A group of 60 people was formed for the intervention group in the WhatsApp app. After each session, educational videos, clips, pamphlets, and tips were provided to them. Throughout this period, the control group was provided with the usual training and care within the healthcare system. Three and six months after the end of the educational interventions, two groups completed questionnaires based on BRT constructs. Additionally, periodic examinations were conducted, including fasting blood sugar checks and monitoring of HbA1c levels of the patients. Individuals were also provided with notebooks to record their daily blood sugar levels. The educational intervention was designed and implemented based on BRT for the intervention group and was carried out over eight weeks.

For the intervention group, the nutrition consultant and ophthalmologist appointments were arranged based on the people’s needs and the internal specialist’s diagnosis for diet training.

In the first session of our educational intervention, our main goal was to improve patients’ understanding of diabetes, its complications, and self-management behaviors. Participants were invited to attend the session, and we explained the objectives of the meeting to them. We emphasized the importance of understanding diabetes for successful self-management and provided a clear definition of diabetes, an overview of its different types (type 1, type 2, gestational), and discussed the role of insulin in managing blood sugar levels. We also covered common symptoms and risk factors associated with diabetes, as well as potential complications that can arise from uncontrolled blood sugar levels, such as heart disease, kidney disease, and nerve damage. We stressed the importance of prevention through proper management and explained the need for regular monitoring. We also provided information on appropriate methods for checking blood sugar levels using a glucometer or continuous glucose monitor (CGM) and shared recommended target ranges for fasting and postprandial glucose levels. Additionally, we highlighted the role of exercise in controlling blood sugar levels and provided examples of suitable exercises that can be incorporated into daily routines based on individual preferences and limitations. We also discussed diet management techniques, including carbohydrate counting and the plate method approach, and offered practical tips on creating a balanced diet plan focusing on whole grains, proteins, vegetables/fruits/fiber-rich foods while limiting processed sugars, saturated fats, and alcohol intake. Finally, the session included a facilitated group discussion where participants were encouraged to share their experiences or concerns regarding self-management behaviors related to diabetes.

The second session aimed to recap the previous session and emphasize the advantages of self-management behaviors for diabetes. Participants gained an understanding of how important self-management is in preventing severe complications related to diabetes. The session also focused on changing attitudes towards implementing these behaviors and addressing any obstacles that may hinder their adoption. The duration of the session was 60 min. Examples of potential complications associated with poor management were discussed, and a question-and-answer segment encouraged active participation from participants, allowing them to share their experiences, challenges, and successes in managing diabetes. Additionally, a brainstorming activity was conducted where participants identified common obstacles or barriers to implementing self-management behaviors. Strategies and solutions for overcoming these obstacles were discussed, with participants encouraged to share their own tips and techniques for overcoming challenges. In conclusion, key takeaways from the session highlighting the benefits of self-management behaviors in preventing complications were summarized. Additional resources for further learning were provided as well. It’s important to note that PowerPoint slides and images were used to show the chronic complications associated with diabetes. This was done to raise awareness about the potential consequences of poor management and to evoke emotions in their virtual group. We also showed educational video clips featuring real examples of diabetic foot ulcers, amputations, and vision impairment caused by uncontrolled diabetes.

During our third session, we aimed to evaluate our previous meetings, engage in open discussions, and encourage dialogue about the social impact and perspectives of close individuals, doctors, and healthcare workers on self-management behaviors. Our goal was to improve adherence to self-management behaviors by creating a supportive environment where participants could freely express their thoughts and feelings. The session lasted for 45 min, and we followed a structured approach that focused on subjective norms and addressed the concept of social influence with self-management behaviors. We provided examples to illustrate how the opinions of close individuals, doctors, and healthcare workers can influence an individual’s commitment to self-management behaviors. Additionally, we encouraged participants to share their personal experiences with social influence related to their health in the virtual group.

The main objective of the fourth educational session was to review the previous session and enhance perceived behavior control by providing a step-by-step guide on self-management. The goal was to equip participants with the necessary techniques for continuous implementation. The session lasted 45 min. A summary of key points discussed in the previous session was presented, followed by an opportunity for participants to ask questions or seek clarification on any concepts or ideas. The concept of gradually and continuously performing self-management behaviors was introduced, highlighting the advantages of breaking down behaviors into smaller, more manageable steps. Practical methods for implementing self-management behaviors in a step-by-step manner were then presented. To encourage participant engagement, interactive discussions, and brainstorming activities were utilized. Additionally, visual aids such as PowerPoints were introduced to enhance understanding and retention of information in the virtual group. Pamphlets or handouts containing relevant information on self-management behaviors were provided as well in the virtual group.

In the fifth training session, we focused on improving meeting effectiveness by actively engaging participants in conversations and considering their suggestions. We discussed reasons for resistance to self-management behaviors, identified solutions, and provided practical health management suggestions. Participants were split into small groups to discuss specific topics related to self-management behaviors and present their ideas. We also addressed potential obstacles and objections and encouraged active participation from all attendees.

During the sixth session of our educational program, titled “Exploring Solutions to Facilitate Self-Management Behaviors,” our goal was to assist participants in learning strategies to make self-management behaviors easier. This involved teaching methods to facilitate exercise, measure blood sugar, and develop positive attitudes toward implementing these behaviors. The 45-minute session included presentations, Q&A sessions, and a guest speaker who shared their successful experience in managing their disease. To enhance the learning process, we utilized educational aids such as clear pictures depicting successful patients who had effectively implemented self-management behaviors. Additionally, we showed video clips related to our goals during the virtual group.

During the seventh session, the focus was on implementing self-management behaviors and helping participants understand the consequences of their actions. The session lasted for 45 min. Participants actively engaged in discussing the impacts of their behavior on self-management. They were encouraged to share personal experiences and insights, while also highlighting the positive outcomes that can be achieved by effectively managing one’s health. Emotional relief exercises were conducted, using techniques like deep breathing and guided visualization, to help participants release any negative emotions or barriers they may have had towards adopting self-management behaviors. A speech was delivered emphasizing the importance of maintaining a positive attitude towards implementing self-management behaviors and highlighting the benefits that stem from this mindset. Relevant video clips showcasing successful patient stories or demonstrating effective techniques related to self-management were shown in the virtual group.

The 8th session aimed to review the previous sessions and allow participants to express their emotions and opinions. The main focus was to understand the reasons why self-management behaviors can be either beneficial or challenging. Various techniques were used to promote the adoption of these behaviors, such as question and answer sessions, interactive discussions, brainstorming activities, and visual aids like PowerPoint slides, images, and video clips. The entire session lasted for 45 min.

The control group was taught about non-related topics, such as communication skills, time management skills, empathy skills, and self-awareness. After the six-month post-test, the control group received a summary of the teaching points provided to the intervention group.

### Statistical analysis

The data were analyzed using SPSS 26 at a 5% significance level. First, we confirmed the normality of the data using the Kolmogorov-Smirnov test (*P* > 0.05). Then, we used frequency descriptive statistics (mean, standard deviation, percentage, and frequency) and chi-square analysis to report and compare the frequency distribution of participants’ demographic characteristics. Next, we conducted repeated measures ANOVA for both primary and secondary outcomes to compare the pre-post intervention within-group means and used an independent t-test for between-group comparisons.

## Results

In this study, there were 120 participants, with 60 people in each group. Out of these, 84 (70.0%) were women and 36 (30.0%) were men. It’s important to note that 3 people from the intervention group and 4 people from the control group were excluded from the study due to personal reasons and not completing the questionnaire. Ultimately, 113 people took part in the educational intervention sessions and completed the 3-month and 6-month post-test questionnaires. Of the 113 participants, 57 were in the intervention group and 56 were in the control group, and they were aged between 30 and 60 years (M = 54.40, SD = 5.83).

A total of 97 patients (80.8%) were over 50 years old and 75 patients (62.5%) had a family history of diabetes. Additionally, 80 (66.7%) were smokers, while only 37 (30.8%) had primary education. According to Table 1, it was found that there is no significant difference between the control and intervention groups in terms of the frequency distribution of demographic variables (*P* < 0.05).

**Table 1:** Frequency distribution of control and intervention groups according to demographic variable

Variable		Total	Group		*X* ^2^	p
		Percent (%)	Intervention	Control		
Sex	Female	84(70/0)	46(76/7)	38(63/3)		0/111
	Male	36(30/0)	14(23/3)	22(36/7)	2/54	
Family history	Yes	75(62/5)	35(58/3)	40(66/7)	0/89	0/346
	No	45(37/5)	25(41/7)	20(33/3)		
Education	illiterate	35(29/2)	19(31/7)	16(26/7)		0/890
	Elementary	37(30/8)	17(28/3)	20(33/3)		
	Middle school	16(13/3)	7(11/7)	9(15/0)	1/25	
	High school	23(19/2)	13(21/7)	10(16/7)		
	University	9(7/5)	4(6/7)	5(8/3)		
Smoking	Yes	40(33/3)	21(35/0)	19(31/7)	2/04	0/360
	No	80(66/7)	39(65/0)	41(68/3)		

As is shown in Table 2, The study found that the mean scores for all BRT constructs (knowledge, attitude, intention, perceived behavioral control, subjective norms, reasons for, reasons against, and behavior) changed significantly over time and between the intervention and control groups. At the start of the study, there was no significant difference in the mean scores for all constructs between the two groups. However, after the educational intervention, the mean scores in the intervention group showed a significant increase in all BRT constructs except for the reasons against self-management. These changes were seen in the second and third time points. In the pre-test, there was no significant difference in the average levels of HbA1C and FBS between the two groups. However, changes were observed in the average level of HbA1C in the second and third time points between the two groups. After the educational intervention, the intervention group showed a significant decrease in the average levels of HbA1C and FBS compared to the control group.

**Table 2:** Comparing the mean scores of BRT constructs and behavior between the intervention and control groups before and after the intervention

Variable	Time	Intervention mean (SD)	Control Mean (SD)	Time	Group	Time*group
Knowledge	Before	5.2 ± 1.3	5.1 ± 1.2	0.05	˂0.001	0.007
	After (3months)	5.9 ± 0.9	5 ± 1.1			
	After (6months)	5.8 ± 1	5.1 ± 1.1			
Reason for	Before	19.5 ± 2.6	19.2 ± 2.3	˂0.001	˂0.001	˂0.001
	After (3months)	25.2 ± 2.3	19.4 ± 2.1			
	After (6months)	25.0 ± 2.3	19.4 ± 2.3			
Reason against	Before	36.4 ± 3.0	35.0 ± 3.4	˂0.001	˂0.001	˂0.001
	After (3months)	27.1 ± 2.2	36.8 ± 3.0			
	After (6months)	27.2 ± 2.2	36.3 ± 2.7			
	Before	23.6 ± 2.4	22.4 ± 2.4	˂0.001	˂0.001	0.028
Subjective Norms	After (3months)	24.8 ± 2.6	27.2 ± 2.7			
	After (6months)	26.7 ± 2.5	26.0 ± 2.9			
Attitude	Before	38.1 ± 5.9	38.3 ± 5.1	˂0.001	˂0.001	˂0.001
	After (3months)	49.6 ± 3.0	36.5 ± 5.6			
	After (6months)	49.5 ± 3.0	37.2 ± 5.4			
	Before	20.4 ± 2.9	19.9 ± 2.6	˂0.001	˂0.001	˂0.001
Perceived Behavioral Control	After (3months)	26.50 ± 2.2	19.7 ± 2.8			
	After (6months)	26.53 ± 2.2	19.5 ± 3.0			
	Before	28.4 ± 2.4	28.5 ± 2.6	˂0.001	˂0.001	˂0.001
Intention	After (3months)	34.1 ± 2.7	28.0 ± 2.2			
	After (6months)	34.1 ± 2.9	28.4 ± 2.3			
Behavior	Before	19.7 ± 1.3	19.6 ± 1.2	˂0.001	˂0.001	˂0.001
	After (3months)	22.5 ± 1.4	19.6 ± 1.3			
	After (6months)	22.4 ± 1.4	19.4 ± 1.2			
	Before	161.02 ± 58.15	153.80 ± 55.26	0.480	0.062	˂0.001
FBS	After (3months)	157.32 ± 62.08	171.07 ± 66.14			
	After (6months)	138.72 ± 54.27	180.27 ± 57.03			
	Before	7.73 ± 1.76	7.52 ± 1.53			
HbA1C	After (3months)	7.27 ± 1.48	7.83 ± 1.62	0.503	0.004	˂0.001
	After (6months)	6.52 ± 1.13	8.54 ± 1.75			

## Discussion

The current study focuses on diabetes self-management education in patients with diabetes and the effect of the DSME program on diabetes knowledge and self-management activities of diabetic patients with the help of the behavioral reason theory.

This study showed that diabetic self-management education patients based on behavior reason theory promotes preventive behaviors of complications related to diabetes and improves the control of FBS and HbA1C in patients, and it was able to improve the level of knowledge (awareness), attitude, and performance of patients. It has a positive effect and by increasing the reasons for positive behavior, it was able to improve people’s motivation and intention and their ability to adopt healthy behaviors.

The study found that the average knowledge score increased in the intervention group after educational interventions were implemented. This is consistent with the findings of Hosseini et al. [32]. One of the fundamental requirements for behavior change is an increase in knowledge, particularly when it comes to health behaviors [33]. Studies evaluating the knowledge of diabetic patients have confirmed the importance of educating patients about their disease. Insufficient knowledge among patients is ineffective in promoting self-care and preventing diabetes complications [34]. Previous research has shown that increased awareness and knowledge positively affect patients’ ability to manage their disease, self-care, reduce blood sugar levels, and control their diabetes [35, 36].

In a study conducted by Debussche et al., it was found that peer-led diabetes education provided once in every three months for a year to patients with type 2 diabetes did not lead to significant improvement in their diabetes knowledge score [37]. This finding is consistent with the present study’s results. Additionally, in Dan et al.‘s study, a 10-minute media training did not improve patients’ awareness [38]. One possible reason for the failure of the program could be the one-sided and short duration of the training or the lack of acceptance of training and change by people.

The results of the study indicate that the intervention group had a higher mean score in their attitude toward self-management behaviors after receiving educational interventions. This suggests that people were more likely to adopt and continue these behaviors when they believed they had positive effects on their health. This finding is consistent with previous research that has shown a positive correlation between attitudes toward self-management behaviors and their adoption [39]. The results of this study are consistent with the findings of Sadeghi et al.‘s study [40]. However, in Khalaf et al.‘s study [41], people’s attitudes did not change, which could be due to the short duration of the study. Unlike awareness, changing attitudes requires longer interventions. Besides, the study conducted in London by Zwarenstein et al. [42] showed that training doctors by providing educational booklets to improve their attitude towards referring patients to ophthalmology examinations was ineffective. This study contradicts the present study, indicating that providing educational pamphlets should not be the only way to educate and change attitudes, even though it is a low-cost method.

The study found that the intervention group experienced a significant increase in their average subjective norms score after receiving educational interventions. As a result, people were better able to understand the importance of social support in adopting healthy behaviors and reducing risky behaviors related to their disease. This indicates that patients are more likely to adopt self-management behaviors when they feel pressure from family members, particularly spouses, doctors, healthcare workers, and friends. Similar findings were reported by Babazadeh et al. [39] and Zindatalab et al. [43]in their studies, which showed that providing educational programs for those who influence patients can improve subjective norms and encourage self-care behaviors in patients with type 2 diabetes.

Behavioral reasons “for/against” are among the other constructs of the theory of behavioral reasoning, which, like the construct of obstacles and perceived benefits in the health belief model, can play a vital role in the effects of knowledge on behavior and have a significant effect on The theory of behavioral reasoning includes constructs of “behavioral reasons for/against” that can significantly impact the relationship between knowledge and behavior. Similar to the constructs of “obstacles” and “perceived benefits” in the health belief model, behavioral reasons can affect a person’s attitude and performance, resulting in a behavior change. Studies [44, 45] have shown that after educational interventions, the mean score of favorable behavioral reasons has increased while the score of opposed behavioral reasons has decreased. The reasons for opposing behavior can include physical, psychological, or financial barriers that prevent a person from performing appropriate self-management behaviors. However, the mean score of behavioral reasons against providing appropriate solutions decreased significantly after educational interventions. By increasing the reasons for favorable behavior after the implementation of educational interventions, it is possible to increase the motivation and willingness of patients to implement self-management behaviors and adhere to them.

Our study results demonstrated that the intervention group’s average intention score increased post the implementation of educational interventions. These interventions can play a crucial role in enhancing diabetes self-management behaviors by increasing people’s intentions. Higher intention levels can lead to faster behavior implementation, and our study’s findings are consistent with a similar study by Damayanti and colleagues [46]. However, our results do not support Robin et al.‘s study, which suggests that only 50% of intention can influence behavior implementation [47].

Compared to the mean score of self-management behavior, three and six months after the intervention, there was a significant increase in the intervention group. Based on the results of the present study, increasing awareness as well as other constructs of the behavioral reason theory, including attitude, behavioral intention, and perceived behavioral control, all led to an increase in the skills and performance of individuals and improved self-management behaviors of the intervention group.

The results of our study demonstrated that the educational intervention based on behavioral reasoning theory resulted in significant improvements in HbA1C levels in the intervention group compared to the control group. Lowering HbA1C levels helps reduce the risk of developing diabetes-related complications such as cardiovascular diseases, kidney problems, and nerve damage [48]. The HbA1C test is valuable in diagnosing diabetes and assessing glycemic control. It provides accurate results and is easy to administer, making it particularly useful in low- and middle-income countries. This information can guide treatment decisions and help prevent uncontrolled blood sugar-related complications [48]. Recent studies have shown that Diabetes Self-Management Education (DSME) leads to a moderate decrease in HbA1c compared to usual care for people with type 2 diabetes, regardless of the treatment method used [49, 50]. Other studies have also demonstrated that education can improve self-care variables, fasting blood sugar control, and HbA1c levels [51]. A study conducted by Zheng et al. [52] showed that self-management training of diabetic patients improved the fasting blood sugar and HbA1c results in the intervention group, which supports the findings of the present study. Therefore, healthcare workers are recommended to teach diabetic patients how to implement self-management behaviors to achieve better blood sugar control.

Previous studies have demonstrated that educating and intervening with type 2 diabetic patients can effectively improve their performance and behavior, which is consistent with the results of the current study [40, 53]. This highlights the importance of targeted education and support to empower individuals with type 2 diabetes to manage their condition effectively. Overall, these results reinforce the importance of comprehensive diabetes self-management education programs incorporating behavioral reasoning theory principles for improving blood sugar control outcomes. They emphasize that providing individuals with knowledge, skills, attitudes, intentions, and support can positively change their self-management behaviors and ultimately result in better glycemic control over time.

## Strengths and limitations

No study has been conducted on this topic, and the present study is the first to research this area, which is one of the strengths of this study. It is recommended to utilize this theory and focus on the behavioral reasons for “yes/no” in the self-management education of diabetic patients and other chronic diseases in the future. In this research, the subjects in the control and intervention groups were selected through random sampling, and the research lasted for six months. It is also suggested to evaluate this educational method in patients with type 1 diabetes.

Due to the limited sample in this study, we did not compare self-management behaviors, blood glucose control, and educational intervention effects in different types of antidiabetic treatment (such as insulin injections versus oral medications).

The study was conducted in a specific population of adults with Type 2 diabetes in Bushehr, Iran. Therefore, the findings may not apply to other populations with different demographic characteristics or healthcare systems. Furthermore, this study measured diabetes control indicators such as Fasting Plasma Sugar (FBS) or HbA1c levels. These measurements are important for evaluating overall diabetes management and could provide additional insights into how self-management interventions impact diabetes control outcomes. Since subjects’ willingness was considered to participate in the study, the selection bias was not avoidable and these results cannot be generalized to all diabetes patients.

## Conclusion

Diabetes self-management education, with the help of behavioral reasoning theory, can effectively improve the level of self-management performance in patients with type 2 diabetes. This can be achieved by creating a positive attitude and improving the mental norms of patients towards the implementation of self-management behaviors, which in turn leads to better blood sugar control. This educational program can serve as a useful model for promoting health-related behaviors.

The findings of this study have significant implications for healthcare practice, policy development, and future research in the field of diabetes self-management education. In terms of practice, the results highlight the effectiveness of the DSME program based on behavioral reason theory in enhancing diabetes knowledge and self-management behaviors among patients. Therefore, healthcare providers should consider integrating this educational intervention into their standard care for individuals with diabetes to improve disease management and control.

From a policy perspective, it is crucial to invest in comprehensive diabetes education programs that go beyond knowledge acquisition and encompass attitudes, intentions, and perceived behavioral control. Policymakers should prioritize funding for these programs to ensure that individuals with diabetes receive adequate support and resources to effectively manage their condition. By allocating resources towards training healthcare professionals on delivering the DSME program or establishing partnerships with community organizations, policymakers can reach underserved populations more effectively.

In terms of future research directions, conducting long-term follow-up studies would be valuable to assess the long-lasting impact of the intervention on knowledge retention, self-management behaviors, and clinical outcomes. This will provide insights into whether behavior change is sustained over time among participants who received the DSME program. Additionally, exploring alternative delivery methods such as online platforms or mobile applications could enhance accessibility and enable reaching a wider range of individuals who may face barriers to accessing traditional face-to-face education.

Overall, this study underscores the importance of implementing effective educational interventions based on behavioral reason theory in both clinical practice and policy initiatives targeting individuals with type 2 diabetes. By addressing key factors such as knowledge enhancement along with attitudes, intentions, and perceived behavioral control through comprehensive education programs, promoting better disease management outcomes becomes possible for individuals living with diabetes.

### Electronic supplementary material

Below is the link to the electronic supplementary material.

Supplementary Material 1

Supplementary Material 2

## Data Availability

Data used in the analysis as well as all programs used for the analysis may be obtained by contacting the corresponding author on reasonable request.
