# Supplementary material for: The effect of educational intervention based on the behavioral reasoning theory on self-management behaviors in type 2 diabetes patients: a randomized controlled trial
Source: BMC Public Health. 2024 Jul 2;24:1761. doi: 10.1186/s12889-024-19207-0 (PMC11218263; doi:10.1186/s12889-024-19207-0)
Supplement: Supplementary file 2 — Supplementary Material 2 [file 12889_2024_19207_MOESM2_ESM.docx]

**The Persian version of this questionnaire was used in this study**

**Demographic information**

**How old are you?** ……………….. years

Sex:

**Your marital status:** 🞎 Single 🞎 Married 🞎 Divorced

**Your job status:** 🞎 Employed Full time 🞎 Employed part-time Unemployed

**Do you smoke**: 🞎 Yes 🞎 No

**The history of diabetes in the family:** 🞎Yes 🞎No

**Education:** 🞎Illiterate 🞎elementary 🞎Middle school 🞎 High school 🞎 university

**Behavior questionnaire**

**Scale: Never, Seldom, Occasionally, Always**

1. I exercise daily for at least 30 minutes, five times a week.

2. I undergo periodic examinations every month to monitor my health.

3. I am diligent in taking my blood sugar medication on time.

4. I regularly check and examine my feet to ensure no issues.

5. I measure my blood sugar levels daily to stay on top of any changes or concerns.

6. I follow the diet recommended by my doctor to manage my condition.

**The following questions are related to knowledge of diabetes, please select the correct answer.**

**Scale: (1 = yes, 0 = no)**

1. High blood sugar levels can increase the risk of a heart attack.

2. Exercise does not directly regulate blood sugar levels.

3. High blood sugar can cause damage to the kidneys.

4. Regular medical check-ups can help prevent the progression of the disease.

5. It is important to regularly examine and care for your feet.

6. Regular visits to the doctor are not important.

7. Following the recommended diet from your doctor is essential for controlling blood sugar levels.

**The following questions are based on BRT constructs in the field of implementing self-management behaviors**

**Scale: (I completely disagree=1, I completely agree=5)**

1. I feel that adhering to a proper diet will help protect me from the potential complications of diabetes.

2. I think that monitoring my blood sugar levels daily is an effective way to control my blood sugar.

3. I think that engaging in daily exercise will help me manage my diabetes.

4. I feel that regular check-ups at the health center, such as monthly examinations, are very beneficial for me.

5. I believe that taking care of my feet and skin can prevent foot and toe ulcers.

6. I trust that following my doctor's advice will assist me in controlling my blood sugar levels.

7. I think that by following a diabetic-specific diet, I can reduce my concerns about the progression of my disease.

8. I believe that scheduling regular appointments with my doctor is crucial to managing my blood sugar and preventing it from worsening.

9. I think that by successfully managing my blood sugar levels, I may no longer need to continue taking certain medications.

10. I believe that it is not necessary to have regular check-ups, such as monthly examinations, to monitor and manage my diabetes effectively.

11. I think trying to control blood sugar is useless because in any case, complications (kidney, eye, skin, and ulceration) of the disease occur.

12. I think diabetes is chronic and lifelong.

13. I plan to exercise daily (30 minutes of walking, at least 5 times a week).

14. I decided to do my periodical examinations regularly every month.

15. I decide to take my blood sugar medication on time

16. I plan to take care of my feet regularly.

17. I decide to measure my blood sugar daily.

18. I decide to follow my doctor's advice on diet

19. I decide to eat all my favorite foods regardless of my illness.

20. I decide to see my doctor regularly even if my blood sugar level is normal.

21. I decide to continue taking my blood sugar medication even if my daily blood sugar levels are normal.

22. It is hard for me to exercise (walking) daily

23. I find it easy to take care of my feet.

24. I cannot adjust the intake of my blood sugar medication with the time of eating

25. It is difficult for me to follow my doctor's orders and follow his recommendations at home.

26. It is difficult for me to follow the diabetic diet with my family members.

27. It is easy for me to go to the health center for periodical examinations.

28. It is difficult for me to make an appointment to see my doctor

29. My friend's opinion about my diet is important to me.

30. My partner encourages me to take necessary steps to control my blood sugar, such as exercising regularly, monitoring my blood sugar levels, having regular checkups, following a healthy diet, taking care of my feet, and seeing my doctor.

31. My friend encourages me to make the necessary recommendations to control my blood sugar (doing regular exercise, measuring my blood sugar, periodic examinations, observing my diet, taking care of my feet, and visiting my doctor).

32. My doctor encourages me to make the necessary recommendations to control my blood sugar (do regular exercise, measure my blood sugar, have regular check-ups, follow my diet, take care of my feet, and visit my doctor).

33. My doctor expects me to follow my diet all the time.

34. My doctor believes that I should do periodic examinations regularly (monthly).

35. The healthcare workers expect me to go to the health center every month for periodical examinations.

36. Measuring the regularity of my blood sugar prevents the progress of my disease

37. Adhering to the diet helps a lot to control my blood sugar

38. Periodic examinations can prevent the long-term complications of my disease

39. Foot/skin care prevents the formation of wounds and is effective in preventing the complications of foot injuries

40. Visiting my doctor will help me control my blood sugar better

41. Exercising is effective in preventing complications and progression of my disease

42. It is unpleasant for me to take my medicines regularly

43. I don't have the conditions to do sports

44. It bothers me to follow a diet because food is expensive

45. Performing periodical (monthly) examinations is time-consuming

46. It is not possible for me to see a doctor regularly because of my financial situation and the expense of the visit

47. I don't have enough information about diabetic diet

48. Getting a blood sugar measuring device is expensive for me

49. I do not have access to the health center for periodical examinations

50. I don't know how to inject insulin.

51. It bothers me to measure blood sugar regularly
